# Potential of Dried Blood Self-Sampling for Cyclosporine C_2_ Monitoring in Transplant Outpatients

**DOI:** 10.1155/2010/201918

**Published:** 2010-06-27

**Authors:** Alexander Benedikt Leichtle, Uta Ceglarek, Helmut Witzigmann, Gábor Gäbel, Joachim Thiery, Georg Martin Fiedler

**Affiliations:** ^1^Institute of Laboratory Medicine, Clinical Chemistry and Molecular Diagnostics, University Hospital Leipzig, 04103 Leipzig, Germany; ^2^Surgical Clinic and Policlinic II, University Hospital Leipzig, 04103 Leipzig, Germany

## Abstract

*Background*. Close therapeutic drug monitoring of Cyclosporine (CsA) in transplant outpatients is a favourable procedure to maintain the long-term blood drug levels within their respective narrow therapeutic ranges. Compared to basal levels (C_0_), CsA peak levels (C_2_) are more predictive for transplant rejection. However, the application of C_2_ levels is hampered by the precise time of blood sampling and the need of qualified personnel. Therefore, we evaluated a new C_2_ self-obtained blood sampling in transplant outpatients using dried capillary and venous blood samples and compared the CsA levels, stability, and clinical practicability of the different procedures. *Methods*. 55 solid organ transplant recipients were instructed to use single-handed sampling of each 50 *μ*L capillary blood and dried blood spots by finger prick using standard finger prick devices. We used standardized EDTA-coated capillary blood collection systems and standardized filter paper WS 903. CsA was determined by LC-MS/MS. The patients and technicians also answered a questionnaire on the procedure and sample quality. *Results*. The C_0_ and C_2_ levels from capillary blood collection systems (C_0_ [ng/mL]: 114.5 ± 44.5; C_2_: 578.2 ± 222.2) and capillary dried blood (C_0_ [ng/mL]: 175.4 ± 137.7; C_2_: 743.1 ± 368.1) significantly (*P* < .01) correlated with the drug levels of the venous blood samples (C_0_ [ng/mL]: 97.8 ± 37.4; C_2_: 511.2 ± 201.5). The correlation at C_0_ was *ρ*__cap.-ven.__ = 0.749, and *ρ*__dried blood-ven__ = 0.432; at C_2_: *ρ*__cap.-ven.__ = 0.861 and *ρ*__dried blood-ven__ = 0.711. The patients preferred the dried blood sampling because of the more simple and less painful procedure. Additionally, the sample quality of self-obtained dried blood spots for LC-MS/MS analytics was superior to the respective capillary blood samples. *Conclusions*. C_2_ self-obtained dried blood sampling can easily be performed by transplant outpatients and is therefore suitable and cost-effective for close therapeutic drug monitoring.

## 1. Introduction

Despite the upcoming new drugs, Cyclosporine A (CsA) is still one of the most important immunosuppressants in solid organ transplantation. Due to its narrow therapeutic range, the high intra- and interindividual variability of absorption and metabolization, and the need of highly compliant daily administration, long-term accurate and frequent monitoring of CsA concentrations is pivotal to avoid graft rejection at underdose and nephrotoxicity at overdose. Since the variation in CsA exposure is greatest during the absorption phase in the first 4- to 12-hour post-dose, the determination of the pharmacokinetic area under the curve (AUC_0–4_, resp., AUC_0–12_) [1, 2] provides an adequately precise measure of drug exposure. However, AUC determination requires multiple blood samplings; it is uncomfortable for the patient, expensive, and difficult to perform in a routine clinical setting. The commonly performed measurement of predose CsA concentrations (C_0_) is, in contrast, less applicable for CsA pharmacokinetic monitoring since it does not perfectly correlate with CsA exposure as determined by AUC analysis, and it does not predict nonoccurrence of graft rejection [2]. In contrast, the CsA C_2_ (2 hours post-dose) peak concentration highly correlates with AUC. Therefore, CsA C_2_ levels have been initially described as the optimal single-time point marker for AUC [3–5]. However, C_2_ monitoring bears some practical difficulties due to the narrow time frame of ±15 min for blood sampling performed by qualified medical personnel. Hence, it is reasonable to shift the sampling work to capable patients who can profit from the enhanced sampling accuracy, even when they stay at home [6]. Novel mass spectrometric-based analytical methods for the determination of CsA only require small volumes of EDTA-whole blood (≤50 *μ*L). Therefore, self-sampling systems like capillaries or dried blood spots become applicable for transplant patients. Our paper aimed at the evaluation and comparison of feasible CsA C_2_ self-sampling procedures for capillary EDTA and dried blood in patients after solid organ transplantation with respect on reproducibility, accuracy, sampling quality, and particularly the patient-related practicability and acceptance.

## 2. Methods and Materials

### 2.1. Patients

55 solid organ transplant recipients (42 renal, 2 combined renal/pancreas, 11 liver transplants; m/f 37/18; age 52 years ±10) from the outpatient clinic of the Transplant Center of the University Hospital of Leipzig were recruited for this paper. All patients received daily CsA dosing (120–500 mg/d), 42 patients additionally MMF and were on steady state medication at least three months after transplantation.

 The study was approved by the local ethics committee and fulfilled all requirements of the latest amendment of the Helsinki declaration. All patients declared their informed consent.

### 2.2. Study Design

The study included three visits, number 1 and number 3 at the clinic, number 2 at home. Visit number 1 comprised patients recruitment and training for standardized self-sampling of capillary EDTA-whole blood and dried blood, venous blood drawing, and exemplary sampling of capillary blood and dried blood, as well as delivering a prepared kit for home sampling. At visit number 2, about four weeks after visit number 1, patients obtained capillary blood and dried blood at home and shipped it to the laboratory using the prepared kits. Visit number 3 consisted of venous sampling and supervised capillary self sampling as well as filling out the questionnaire. Sampling quality of all visits was controlled before analysis using a standardized checklist. Blood specimens were assessed in the lab. by the technicians with respect to sample quality.

### 2.3. Material

For capillary sampling we used mechanical finger-prick devices (Accu-Check Softclix Pro, Roche Diagnostics, Mannheim, Germany), EDTA-coated capillary vials (Microvette No 20.1278, Sarstedt, Nümbrecht, Germany), and specimen collection filter paper (Whatman & Schüll No 903, Whatman, Middlesex, UK). For dried blood spots, capillary EDTA-blood was dropped on the filter paper and air dried thereafter for at least 2 h. Systems for venous blood sampling (EDTA Monovette and Multifly needle sets) were obtained from Sarstedt (Nümbrecht, Germany). Postpaid shipping kits pursuant UNO “UN 3373” norm were prepared for the patients.

### 2.4. Analytical Methods

We determined CsA levels by liquid chromatography tandem mass spectrometry (LC-MS/MS) in venous and capillary EDTA-blood (50 *μ*L sample volume) as previously described [7]. For the CsA analysis in dried blood a 4 mm diameter spot (corresponding blood volume 4 *μ*L) was eluted with 100 *μ*L methanol containing Cyclosporine D (CsD) as internal standard. After stirring (20 min), CsA measurements were performed using 35 *μ*L of the supernatant.

### 2.5. Patient Questionnaire

The patient-related practicability was assessed by the following questions: Could you draw capillary blood without help? If not, when did you need help? Do you prefer capillary or venous sampling? Do you prefer capillary EDTA vials or specimen collection filter paper? Did you encounter problems shipping the samples?

### 2.6. Technician Questionnaire

Sampling quality was assessed by the following criteria: sample volume, observed clotting, proper filter, and paper dropping.

### 2.7. Statistics

Statistical analyses were performed using MedCalc (Mariakerke, Belgium), SPSS (Chicago, USA), and R [8] with the latticist package [9]. For correlation analysis, Spearman's *ρ* coefficient was calculated. Significance of differences between groups was computed with the Mann-Whitney-*U*-test [*(*P* < .05); **(*P* < .01)], normal distribution was ensured using the one-sided Kolmogorov-Smirnov test.

## 3. Results

### 3.1. CsA Concentrations in Capillary Blood Correlate with Venous Levels

CsA C_0_ and C_2_ concentrations in venous blood correlated significantly with concentrations obtained from capillary EDTA blood and to a lesser but also significant extent with CsA concentrations derived from dried blood spots (*Table 1*).

### 3.2. CsA Is Stable in Dried Blood Spots at 8°C

**Figure 1:**
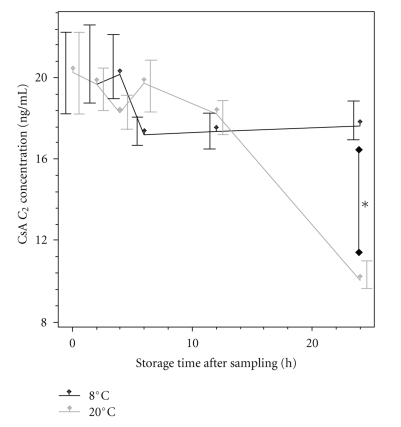
Stability of CsA in dried blood spots at different ambient temperatures. Medians and 95% coverage intervals of effective CsA C_2_ concentrations in dried blood spots stored at 8°C and 20°C, *(*P* < .05).

18 capillary dried blood samples with and without EDTA-stabilizing were stored at 8°C and 20°C for 2, 4, 6, 12, and 24 hours and analyzed thereafter (Figure 1). Samples were stable up to at least 12 hours, a significant difference (*P* = .013) in stability between cooled and noncooled dried blood spots aroses, though, only at the 24 h time point. The 24 h total decrease in CsA concentrations was significant in both groups (cooled: *P* < .05; noncooled: *P* < .001). While EDTA failed to exert stabilizing effects in dried blood spots, neither did we find remarkable decomposition of CsA in capillary EDTA blood samples nor is it mentioned in the recent literature [10, 11].

### 3.3. CsA Concentrations Depend on the Sample Material

**Figure 2:**
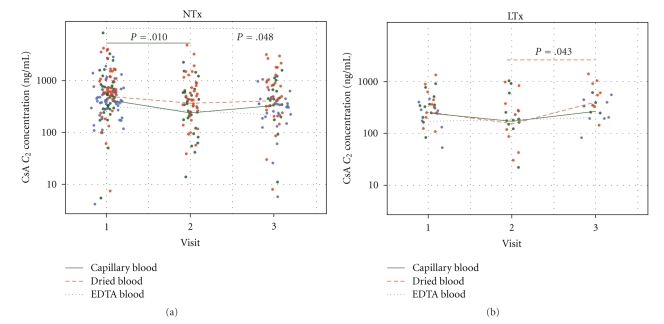
Absolute CsA C_2_ concentrations at all visits. Dotplots of absolute CsA C_2_ concentrations at all visits for renal ((a), including renal/pancreas) and liver (b) transplant recipients. Significant differences are highlighted in the respective colour (capillary blood = green, dried blood = red, EDTA blood = blue). Lines represent medians.

CsA measurement in capillary blood resulted in significantly higher CsA C_0_ and C_2_ levels compared to venous blood. Dried blood spots yielded the highest concentrations and variance (Table 2 and Figure 2). Based on the high correlation to the venous concentrations and the linearity of increase, the discrepancy might be overcome by the introduction of a correction factor. We found a significant (*P* = .010) difference between CsA C_2_ concentrations in capillary blood when comparing the withdrawals performed by a physician (visit 1) and by the patient at home (visit 2) in renal transplant recipients, probably due to the awkward handling of the tubes. In liver transplant recipients, there was a barely significant (*P* = .043) difference between the CsA C_2_ concentrations from self-obtained dried blood samples at visit 2 and from samples under supervision drawns at the transplant center at visit 3. We found also a slight decrease (*P* = .048) of CsA C_2_ concentrations in EDTA blood from visit 1 to visit 3 in renal transplant recipients (Figure 2).

### 3.4. Self-Sampling of Capillary Blood Is Feasible and Appreciable for Transplant Patients

All patients considered themselves to be sufficiently informed to perform capillary sampling at home. 91% of the patients were able to obtain blood without help. 61% prefered capillary sampling to venous sampling, 30% were undecided. 65% versus 18% prefered capillary dried blood to capillary EDTA sampling due to facilitated handling. All patients drew capillary blood from the finger pad, 13% encountered shipping problems caused by the size of the shipping box pursuant to UN 3373. The sample quality of the capillary samples was evaluated by the lab technicians using a predefined checklist. 83% of the dried blood spots and 73% of the self-obtained capillary EDTA samples were adequate, 16% versus 23% of the samples provided insufficient material, and 1% versus 4% was coagulated, respectively.

## 4. Discussion

We observed adequate correlation of C_0_ and C_2_ levels derived from capillary dried whole EDTA blood and the capillary EDTA blood with the corresponding venous blood samples as also stated by Keevil and Merton for the latter [10, 12]. The weaker correlation of CsA C_0_ and C_2_ levels measured in dried blood might be caused by the small sample volume used for analysis (4 *μ*L verus 50 *μ*L EDTA blood), which results in effective CsA concentrations below 10 ng/mL in the processed sample. In this low concentration range CsA contaminations of the internal standard CsD interfere with the measurement [13]. Additionally, the blood volume in the 4 mm dried blood spot varies depending on the individual hematocrit. Moreover, chromatographic effects of CsA (radial concentration gradient and increase of variance) in the filter paper were observed [14]. Therefore, dried blood should only be punched from the center of the blood spot to restrain preanalytical variance. 

 Self-sampling may become an alternative of venous blood taking in monitoring the steady state of CsA immunosuppression especially of outpatients. However, important prerequisites should be kept in mind.

First, self-sampling should be performed in a highly standardized way—thorough instruction and equivalent training of the patients are indispensable. In our study, all patients considered themselves as sufficiently informed and managed the sampling procedure—even without help—very well as expressed by the high percentage (83% and 73%, resp.) of samples suitable for analysis. The main problems were inadequate sample volume (spots less than 4 mm in diameter, or air accidentally included in the capillary tube and preventing further filling) and coagulation, especially in the capillary EDTA systems. Since self-sampling is greatly facilitated using filter paper and is performed with favourable sample quality, patients prefer this technique to the more complicated handling of capillary EDTA vials, which were used in the studies of Keevil, Yonan, and Merton [10–12].

 Second, the analytical sensitivity of the dried blood measurement of low-dose- CsA C_0_ levels can be enhanced by the use of deuterated standards, which are available now or by simply increasing sample volume requiring several blood spots. However, CsA C_2_ concentrations range between 400–1300 ng/mL [1]. Hence, the use of capillary dried blood is feasible for these patients.

 Third, cool and rapid shipping is essential to restrain CsA degradation. While 5-day stability of CsA in capillary EDTA samples was shown by Yonan et al. [11], in our study we could corroborate CsA stability only in dried blood spots kept at 8°C, whereas samples degraded after one day when kept at room temperature (20°C, Figure 1). Interestingly, EDTA did not exert any stabilizing effect on CsA in dried blood spots. This degradation is a challenge for the sample transport management demanding either same-day delivery or warranting refrigerated transport and storage. However, in contrast to capillary EDTA blood the dried blood samples are very convenient for shipping since a simple paper envelope can be used instead of a larger, inexpedient, and more expensive box. 

 Finally, reasonable correction factors should be applied to maintain comparability with venous levels. As shown in Figure 2, the self-obtained CsA C_2_ concentrations and the undersupervision obtained ones correspond very well and indicate that the self-monitoring performed by patients at home is comparable to the monitoring performed at specialized centers. The slightly significant difference in dried blood CsA C_2_ concentrations in liver transplant recipients between visits 2 and 3 was not seen in renal transplant recipients and might point at a still not sufficient training of the patients. Together with the linearity of the increase, which may be adjusted by the introduction of correction factors, capillary blood (EDTA blood or dried blood) was proved to be an appropriate medium for steady-state CsA monitoring in solid organ transplant patients [10–12].

 Additionally, capillary blood samples could also be used to determine further immunosuppressant drugs, ranging from tacrolimus, sirolimus, and everolimus to MMF [6, 15, 16] or even creatinine as a correlate of renal transplant function and nephrotoxicity in renal and nonrenal transplant recipients [12] in a single sample, which renders the method favorable for pediatric transplant patients, where sampling is frequently difficult and cumbersome. From an economical point of view, self-sampling at home can save expenses for qualified ward personnel and travelling costs to the outpatient wards. Additionally, the patient's CsA monitoring results might be already available when the patient undergoes his routine checkup.

 Comparing our results for CsA C_2_ monitoring using capillary EDTA blood with previous studies reveals analogue correlations with the results of the venous “gold standard” method [10, 11]. However, there is only one publication by Azevedo et al. investigating the feasibility of CsA C_2_ monitoring in dried blood, demonstrating a comparable correlation of *r* = 0.81 for radioimmunological assays of dried blood CsA C_2_ concentrations and routine CsA C_2_ determinations from venous whole blood though applying a five times higher dried blood volume of 20 *μ*L [17].

 Apart from the aforementioned advantages of CsA C_2_ montoring using capillary blood, there are several limitations to be considered. The main constraint is the small sample volume, especially favoured in pediatric patients, restricting the number of parameters determined simultaneously and demanding multiparametric methods. Moreover, the capillary sample material is not suitable for many, in particular for the hemolysis-sensitive parameters. The finite stability of the analytes in dried blood might as well interfere with the clinical use of dried blood monitoring as the elaborate analytical methods.

 Taking the above-mentioned factors into account, CsA self-monitoring using capillary EDTA blood or capillary dried blood spots is feasible and comfortable for transplant patients—even at home—with respect on sufficient comparability to venous blood, accuracy for clinical use, sampling quality, and patient-related practicability.

## 5. Conclusion

C_2_ self-obtained blood sampling using EDTA-stabilized capillary and dried blood spots can be easily performed by transplant recipients and results in CsA measurements acceptable for steady-state monitoring. The type of blood sampling influences the respective CsA levels. However, the patient-related intraprocedural variances of CsA measurements are still appropriate for C_2_ monitoring in clinical practice. Therefore, dried blood self-sampling is suitable for Cyclosporine C_2_ monitoring in transplant outpatients.

## Figures and Tables

**Table 1:** Correlations of venous and capillary CsA concentrations.

Visit	Correlation	C_0_	C_2_
(1)	V_E_–C_E_	0,749**	0,854**
	V_E_–C_DB_	0,432**	0,753**
	C_E_–C_DB_	0,521**	0,642**

(2)	C_E_–C_DB_	0,498**	0,835**

(3)	V_E_–C_E_	0.861**	0,861**
	V_E_–C_DB_	0.383*	0,711**
	C_E_–C_DB_	0.274	0,652**

Correlations as Spearman's *ρ*; *(*P* < .05); **(*P* < .01); Venous EDTA blood V_E_; Capillary EDTA blood C_E_; Capillary Dried Blood C_DB_.

**Table 2:** Absolute mean venous and capillary CsA concentrations of all patients at all visits.

Visit	Mat.	C_0_ (*μ*g/L)	C_2_ (*μ*g/L)
(1)	V_E_	95.1 ± 28.7	586.0 ± 247.2
	C_E_	117.4 ± 49.0	710.3 ± 386.5
	C_DB_	171.4 ± 92.6	756.2 ± 378.6

(2)	C_E_	115.7 ± 99.5	507.4 ± 259.7
	C_DB_	146.5 ± 83.2	621.9 ± 359.1

(3)	V_E_	97.8 ± 37.4	511.2 ± 201.5
	C_E_	114.5 ± 44.5	578.2 ± 222.2
	C_DB_	175.4 ± 137.7	743.1 ± 368.1

Absolute CsA C_0_ and C_2_ concentrations at all visits; Venous EDTA-Blood V_E_; Capillary EDTA blood C_E_; Capillary Dried Blood C_DB_.

## Abbreviations

CsA: Cyclosporine A
LC-MS/MS: Liquid chromatography/-tandem mass spectrometry
MMF: Mofetil Mycophenolate
EDTA: Ethylene diamine tetra-acetate
AUC: Area under the curve
C_0_: CsA baseline concentration
C_2_: CsA 2-hour post-dose concentration.
